# Metformin: current clinical applications in nondiabetic patients with cancer

**DOI:** 10.18632/aging.102787

**Published:** 2020-02-18

**Authors:** Kailin Chen, Yajun Li, Zhen Guo, Yong Zeng, Wei Zhang, Hui Wang

**Affiliations:** 1 Key Laboratory of Translational Radiation Oncology, Hunan Province, Department of Radiation Oncology, Hunan Cancer Hospital and The Affiliated Cancer Hospital of Xiangya School of Medicine, Central South University, Changsha 410013, Hunan, P.R. China; 2 Department of Lymphoma and Hematology, Hunan Cancer Hospital and The Affiliated Cancer Hospital of Xiangya School of Medicine, Central South University, Changsha 410013, Hunan, P.R. China; 3 Department of Clinical Pharmacology, Xiangya Hospital, Central South University and Institute of Clinical Pharmacology, Central South University, Hunan Key Laboratory of Pharmacogenetics, Changsha 410008, Hunan, P.R. China; 4 National Clinical Research Center for Geriatric Disorders, Xiangya Hospital, Central South University, Changsha 410008, Hunan, P.R. China; 5 Translational Medicine Center, Hunan Cancer Hospital and The Affiliated Cancer Hospital of Xiangya School of Medicine, Central South University, Changsha 410013, Hunan, P.R. China; 6 Engineering Technology Research Center for Diagnosis-Treatment and Application of Tumor Liquid Biopsy, Changsha 410013, Hunan, P.R. China

**Keywords:** metformin, cancer, nondiabetic patients, prevention, anticancer

## Abstract

Metformin is one of the most commonly used first-line oral medications for type 2 diabetes mellitus. Multiple observational studies, reviewed in numerous systematic reviews, have shown that metformin treatment may not only reduce the risk of cancer but may also improve the efficacy of cancer treatment in diabetic patients. Recent studies have been conducted to determine whether a similar protective effect can be demonstrated in nondiabetic cancer patients. However, the results are controversial. The potential optimal dose, schedule, and duration of metformin treatment and the heterogeneity of histological subtypes and genotypes among cancer patients might contribute to the different clinical benefits. In addition, as the immune property of metformin was investigated, further studies of the immunomodulatory effect of metformin on cancer cells should also be taken into account to optimize its clinical use. In this review, we present and discuss the latest findings regarding the anticancer potential of metformin in nondiabetic patients with cancer.

## INTRODUCTION

Cancer, a major health problem worldwide, is now the leading cause of death in most areas according to the data reported by the National Cancer Center of China and American Cancer Society [1, 2]. To improve cancer outcomes, a number of established medications with known anticancer properties have been investigated.

Metformin, a semisynthetic biguanide, is derived from the French lilac plant. It has been approved for the treatment of type 2 diabetes mellitus (T2DM) and is also frequently used as an off-label treatment for polycystic ovarian syndrome (PCOS) and metabolic syndrome. It is absorbed within 1-3 hours after oral administration, and 90% is excreted metabolized from the kidneys. Its antihyperglycemic effect is mediated by reducing gluconeogenesis, glucose absorption and hepatic glucose production. Metformin also reduces insulin resistance by increasing peripheral glucose uptake [3]. It is a relatively safe drug with a low risk of lactic acidosis and a mild toxicity related to renal function. The most common side effects of metformin are types of gastrointestinal distress, such as anorexia, nausea, abdominal discomfort, and diarrhea [4]. In addition, vitamin B12 deficiency is not common in clinical practice [5].

In addition to the antihyperglycemic effect, there is growing interest in metformin’s potential benefits in cancer. Multiple observational studies, reviewed in numerous systematic reviews, have shown that metformin treatment may not only reduce the risk and mortality of cancer but may also improve the efficacy of cancer treatment in diabetic patients [6–8]. Overall, cancer incidence and mortality were decreased by approximately 10% to 40% in diabetic patients who used metformin at doses of 1,500–2,250 mg per day [9]. Recently, several studies have also been conducted to determine whether a similar protective effect can be demonstrated in nondiabetic cancer patients or in patients with impaired fasting glucose [10]. In this review, we will present and discuss the latest findings regarding the potential anticancer role of metformin in nondiabetic patients with cancer.

## Metformin plays a potential role in the prevention of cancer in nondiabetic patients

Diabetes and cancer are common and complex diseases and share many risk factors, such as aging, obesity, unhealthy diet, and physical inactivity. Hyperinsulinemia, hyperglycemia, and inflammation may be possible mechanisms between cancer and diabetes [11]. Diabetes is a confounding variable in the development of cancer. It can increase the likelihood of the occurrence of various types of cancer, such as cancers of the colon, rectum, pancreas and liver, compared to nondiabetic patients. The survival rates of cancer patients are greatly affected by glucose abnormalities. Diabetics taking metformin seem to have a lower risk of developing cancer and all-cause mortality than those not treated with metformin [12, 13]. This evidence indicates that metformin might be a candidate drug for the prevention of cancer in patients with diabetes. Because diabetes itself is an independent risk factor for cancer, the treatment of diabetes might reduce this risk. Whether the suppressive effect of metformin on cancer is caused by a direct preventive effect of the drug or is due to the cancer-diabetes association remains unclear. In addition, we also do not know whether it is worth giving metformin to patients without diabetes to prevent cancer, although its role in inhibiting carcinogenesis has been demonstrated in various strains of rodents [14].

Researchers have previously shown that metformin suppresses intestinal polyp growth and azoxymethane-induced colorectal aberrant crypt foci *in vivo* models [15, 16]. Later, a short-term clinical trial confirmed that low-dose metformin (250 mg/day) suppressed the formation of colorectal aberrant crypt foci [17]. These findings suggest a potential role for metformin in the chemoprevention of colon carcinogenesis. Then, a multicenter, double-blind, placebo-controlled, randomized phase 3 trial was conducted to assess the protective effects of metformin on sporadic colorectal cancer in patients with a high risk of adenoma recurrence. The results showed that metformin treatment at the same low dose reduced the prevalence and number of metachronous adenomas or polyps in nondiabetic patients after polypectomy. Metformin has chemopreventive potential against colorectal cancer [18]. Preclinical data showed that metformin significantly reduced the size and number of oral tumoral lesions induced by carcinogen and prevented the conversion from precancerous lesions to squamous cell carcinomas [19]. Moreover, Michael et al. reported that 3 cases of nondiabetic patients with head and neck cancer history who continued to present with multiple dysplastic mucosa. After treatment with metformin 500 mg twice daily, the mucosal lesions showed complete or partial regression and did not require any additional surgeries [20]. In addition, the finding that proliferation in tissue samples was lower when treated with metformin has also been confirmed in women with human epidermal growth factor receptor-2 (HER2)-positive ductal carcinoma in situ [21].

The above research results suggest that metformin is a promising therapy to directly prevent the progression of precancerous disease to carcinoma in nondiabetic patients. Long-term studies involving larger sample sizes, many more institutions and ethnic groups are needed. However, other studies have focused on nondiabetic people with certain risk factors, such as obesity.

Obese postmenopausal women have an increased risk of endometrial cancer. Metformin was shown to hinder estrogen-mediated endometrial proliferation in an *in vivo* animal model of hyperinsulinemia and insulin resistance, which indicates that metformin may be clinically useful for preventing endometrial cancer in obese women [22]. Furthermore, in obesity-driven endometrial cancer patients, short-term preoperative use of metformin at a dosage of 850 mg twice daily decreased cellular proliferation in tumors [23]. A prospective trial also confirmed the effects of daily low-dose metformin (850 mg/d) on the endometrium in women with newly diagnosed endometrial cancer by evaluating changes in serum/tumor biomarkers [24]. Subsequently, a prospective randomized clinical trial was carried out to assess the impact of metformin on endometrial cancer risk and obesity-related biomarkers of endometrial cancer risk in postmenopausal obese women with prediabetes. Metformin at 1700 mg/day showed trends toward positive effects on endometrial cancer risk-related serum markers and body composition [25]. However, whether metformin reduces the risk of endometrial cancer in a nondiabetic population should be evaluated in the future in a more intuitive way. In addition, Kalinsky et al. also studied overweight or obese newly diagnosed breast cancer patients. Their study demonstrated that preoperative use of metformin at 1500 mg daily results in a significant change in a number of proteomic markers reflecting a wide range of oncologic activity in these patients [26]. However, 1000 mg/day metformin treatment was also reported to have a favorable effect on controlling glucose and glycated haemoglobin (HbA1C) levels in obese nondiabetic breast cancer patients relative to placebo and metformin treatment at 500 mg/day [27], and metformin was shown to be more effective than the control in nondiabetic breast cancer patients with a high body mass index (BMI).

Age and ovulation were shown to be correlated with the risk of ovarian cancer. Age-associated ovarian fibrosis was found to occur in murine ovaries and postmenopausal human ovaries [28, 29]. Organ fibrosis was associated with tumorigenesis and metastasis. A recent study has observed that metformin use in postmenopausal women may reverse or prevent fibrosis, indicating that the use of metformin may prevent age-associated ovarian fibrosis, decreasing the risk of ovarian cancer [29]. Although a meta-analysis demonstrated that metformin was significantly associated with a lower incidence of ovarian cancer in patients with diabetes [30], studies on the use of metformin to prevent ovarian cancer in nondiabetic patients are lacking.

Taken together, the results of the above studies indicate that metformin may have a biologically direct impact on endometrial cancer and breast cancer in overweight or obese patients without diabetes and on age-associated ovarian fibrosis in postmenopausal women. More studies are still needed to provide clear and direct evidence to confirm the effect of metformin on preventing the development of cancer.

## The effect of metformin on the prognosis in nondiabetic patients with cancer

### Lung cancer

Lung cancer remains the leading cause of cancer-related mortality despite the development of various novel targeted therapies and immune checkpoint inhibitors [1, 31]. Platinum-based doublet chemotherapy with or without bevacizumab followed by maintenance therapy until disease progression is recommended as first-line therapy for advanced or metastatic non-small-cell lung cancer (NSCLC) with the absence of a targeted oncogenic driver mutation or high programmed death-ligand 1 expression. Therefore, researchers are devoted to finding a safe, effective and economical way to improve the clinical benefits of these patients. In nondiabetic mouse models, metformin could prevent tobacco carcinogen-induced lung tumorigenesis [32]. However, the results of subsequent clinical trials are controversial.

Tumor suppressor enzyme liver kinase B1 (LKB1) mutations may define a specific and more aggressive NSCLC subtype. A previous study demonstrated that a metformin analog, phenformin, could induce apoptosis in LKB1-deficient lung cancer cells [33]. Importantly, metformin synergizes with cisplatin against LKB1-mutated tumors and is also capable of preventing or delaying acquired resistance to cisplatin by reducing the number of tumor-initiating cells [34]. The FAME trial was designed to exploit a fasting-mimicking diet and metformin to improve the efficacy of platinum-pemetrexed chemotherapy in advanced LKB1-inactivated lung adenocarcinoma. The primary assumption of the study was that the combination shall improve median progression-free survival from 7.6 months in historical data with chemotherapy alone to 12 months. Another trial showed that the addition of metformin at a dose of 500 mg once daily to gemcitabine and cisplatin chemotherapy reduced the occurrence of chemotherapy-induced nausea in nondiabetic patients with stage IV NSCLC, but no statistically significant improvements in the objective response rate (ORR), progression-free survival (PFS) and overall survival (OS) were found [35].

A single-arm phase 2 trial enrolled 14 advanced nonsquamous NSCLC patients to evaluate the use of metformin with standard platinum-based chemotherapy [36]. No LKB1/STK11 mutations were identified in this clinical trial. The maximum dose of metformin was 1000 mg twice daily. Metformin was administered at 1000 mg/day in week 1, 1500 mg/day in week 2, and then 2000 mg/day thereafter, in divided doses. Metformin was noted to be safe and well tolerated. The objective response rate was 23%, and median progression-free survival and overall survival were 3.9 months and 11.7 months, respectively. This clinical trial did not include a control group without the use of metformin, and there was no significant difference in clinical outcomes compared to historical control [36]. A prospective clinical trial conducted by Marrone et al. enrolled nondiabetic patients with chemotherapy-naive advanced or metastatic nonsquamous NSCLC and randomized them to groups receiving platinum-based doublet chemotherapy and bevacizumab with or without metformin followed by maintenance therapy with bevacizumab and metformin combined or bevacizumab alone. The dose of metformin during the clinical trial was 1000 mg twice daily. A total of 25 patients were enrolled. This study showed a significant clinical benefit in PFS with the addition of metformin to standard first-line treatment in nondiabetic NSCLC patients [37]. The median PFS was 9.6 months for nondiabetic patients adding metformin and 6.7 months for patients without metformin. The two clinical trials mentioned above were terminated early due to the difficulty of enrollment. Subsequently, a pooled analysis of these two trials was conducted [38]. The median PFS for all patients who received metformin plus platinum-based chemotherapy with or without bevacizumab was 6 months, which shows a significant improvement compared to historical controls of platinum-based chemotherapy regimens alone and is commensurate with recent historical control regimens containing bevacizumab. The median OS for all patients was 14.8 months, which represents an improvement compared to historical controls before the advent of immune checkpoint inhibition for driver mutation-negative patients.

As the therapeutic focus of NSCLC shifts to immunotherapy and the interaction of metformin and the immune system, further investigation into the synergism of immune checkpoint inhibition and metformin is warranted.

For patients with advanced EGFR-mutant NSCLC, a tyrosine kinase inhibitor of the epidermal growth factor receptor (EGFR-TKI) is the standard first-line therapy, but the acquired resistance to EGFR-TKIs appears after a median of 10 months [39]. Previous studies showed that metformin had a synergistic effect in combination with gefitinib in LKB1 wild-type NSCLC cell lines [40]. Furthermore, metformin effectively increased the sensitivity of TKI-resistant lung cancer cells to gefitinib or erlotinib *in vitro* and *in vivo* [41]. He et al. designed a multicenter, phase 2 randomized, double-blinded, and placebo-controlled study to evaluate the safety and efficacy of metformin in combination with gefitinib as a first-line therapy in nondiabetic patients with NSCLC, and recruitment was completed [42]. Unfortunately, the addition of metformin resulted in nonsignificantly prolonged PFS or OS in nondiabetic, previously untreated NSCLC patients harboring EGFR mutations [43]. In addition, the safety and activity of metformin combined with erlotinib as a second-line treatment were also evaluated in nondiabetic NSCLC patients with EGFR wild-type [44]. The recommended dose of metformin was defined as 1500 mg/day when combined with erlotinib, and the preliminary activity of this combination was very encouraging, with a median progression-free survival of 20 weeks, although the number of patients in this trial was small.

Recently, a phase 2 clinical study showed an exciting result that the addition of metformin to standard EGFR-TKI therapy in patients with advanced lung adenocarcinoma significantly improves PFS and OS. A total of 139 patients were randomly assigned to receive EGFR-TKIs or EGFR-TKIs plus metformin (500 mg twice a day) [45]. Moreover, LKB1-positive patients seemed to have a better OS when treated with a combination of metformin and EGFR-TKI therapy than when treated with EGFR-TKIs alone. More phase 3, placebo-controlled studies with larger sample sizes are warranted to confirm these conclusions.

### Breast cancer

Studies showed that metformin 1000 mg/day treatment was more effective at controlling breast cancer-related prognostic factors glucose and HbA1C levels than placebo and metformin 500 mg/day treatments in obese nondiabetic patients with breast cancer [27]. In another randomized control clinical trial, metformin given 850 mg twice daily significantly decreased the number of metastatic cases after 6 months of hormonal therapy [46]. The results of these studies seem to indicate that metformin could bring clinical benefits to nondiabetic patients with breast cancer.

Yam et al. conducted a phase 2 trial to evaluate the efficacy and safety of the combination of metformin, everolimus and exemestane in overweight and obese postmenopausal women with metastatic, hormone receptor-positive, HER2-negative breast cancer. Twenty-two patients enrolled in this trial [47]. Metformin was given 1000 mg twice daily. Unfortunately, the median PFS and OS were 6.3 months and 28.8 months, respectively. The survival outcomes have no improvement compared with previous studies. Although this trial had more heavily pretreated patients and a higher proportion of patients with visceral disease, the ORR was 22.7%, which was higher than that in the historical reported data in a phase 3 clinical trial, suggesting that adding metformin confers a potential benefit. In another randomized control clinical trial, sixty postmenopausal women with hormone receptor-positive locally advanced or metastatic breast cancer randomly received aromatase inhibitor with or without metformin 500 mg twice daily. It also failed to show improved efficacy with the addition of metformin [48]. Whether the dosage of metformin was 1000 mg or 500 mg twice daily, the clinical outcomes did not seem to be satisfactory in hormone receptor-positive patients who received aromatase inhibitors. Moreover, a negative result was also obtained when metformin plus chemotherapy was used as the first-line treatment of HER2-negative metastatic breast cancer compared with chemotherapy alone. In this study, one-hundred and twenty-two nondiabetic patients with HER2-negative metastatic breast cancer were randomized to receive chemotherapy combined with metformin (2000 mg/day) or chemotherapy alone [49]. Metformin also showed no significant effect on ORR, PFS or OS in nondiabetic patients with metastatic breast cancer receiving standard chemotherapy [50].

The effect of metformin combined with targeted therapy in nondiabetic patients with breast cancer was investigated next. The METTEN study demonstrated that the rate of pathological complete response was higher in women patients with HER2-positive breast cancer treated with neoadjuvant chemotherapy plus trastuzumab combined with metformin than in patients treated with chemotherapy plus trastuzumab [51]. However, the trial was closed before the first scheduled interim analysis due to slow recruitment, and the quality of evidence should be interpreted with caution. Overall, from the current research, the application of metformin in nondiabetic patients with breast cancer does not seem to achieve the expected results.

### Prostate cancer

Prostate cancer is the most commonly diagnosed type of malignancy in men, ranking among the top five cancers in mortality worldwide [52]. Patients with localized prostate cancer have a recurrence rate of up to 30% despite definitive local therapy. Researchers are always looking for new neoadjuvant treatments to improve the outcomes, but the results have been disappointing [53]. A single-arm study with a small sample size evaluated the effects of metformin on localized prostate cancer in paired pretreatment and prostatectomy specimens. A reduction in the proliferation marker Ki-67 was observed following metformin therapy at dosage of 500 mg three times a day [54]. A randomized placebo-controlled, double-blinded trial investigating the biological effects of metformin in localized prostate cancer is ongoing [55]. The dose of metformin in this trial increases from 500 mg once a day (day 1–2), to 500 mg twice a day (day 3–4), and 1000 mg twice a day from day 5 onwards for 4 weeks until surgery.

For men with metastatic prostate cancer, the current mainstay of treatment is based on hormonal manipulations. Androgen-deprivation therapy is effective, but the disease eventually becomes castration-resistant, usually within the first year of androgen-deprivation therapy. Inhibiting the acquired resistance or restoring sensitivity to the drugs may be a way to prolong progression-free survival. Therefore, researchers have tried to evaluate the effect of metformin alone or in combination to improve prostate cancer-related outcomes. A multicenter phase 2 trial enrolled forty-four men with progressive metastatic castration-resistant prostate cancer. Patients received metformin 1000 mg twice daily until disease progression. Metformin was safe and showed modest activity, which only had some influence on prostate-specific antigen level in nondiabetic patients [56]. In addition, abiraterone acetate, an androgen signal inhibitor, is one of the preferred first-line treatments in metastatic castration-resistant prostate cancer. As a combination therapy, the addition of metformin to abiraterone for patients with metastatic castration-resistant prostate cancer showed no meaningful clinical benefit. Metformin was also given at 1000 mg twice daily in this study [57]. However, these studies were limited by small sample sizes. A larger trial in which metformin is added to androgen-deprivation therapy in patients with castration-sensitive locally advanced or metastatic patients is recruiting (NCT00268476) [58]. The estimated study completion date will be 2024.

### Endometrioid endometrial cancer

Endometrioid endometrial cancer shows a strong association with obesity and insulin resistance [59]. Preclinical studies demonstrated that metformin reduced proliferation and promoted apoptosis in endometrioid endometrial cancer cells [60, 61]. Some small, nonrandomized, open-label preoperative clinical trials for endometrial cancer found a reduction in cancer cell proliferation, as measured by immunohistochemical expression of Ki-67 in metformin-treated patients [24, 62–64]. Although the usage and dosage of metformin varied from 850 mg daily, 850 mg twice a day, 500 mg three times a day to 2250 mg per day in these trials, the results were consistent with each other. To provide high-quality evidence of an antiproliferative effect of metformin, a placebo-controlled, double-blind, randomized trial was conducted. Eighty-eight women with atypical hyperplasia or endometrioid endometrial cancer were randomized to receive metformin or placebo. Only two patients were diagnosed with diabetes in the placebo group, and the others were non-diabetic patients. However, short-term treatment for 1 to 5 weeks until surgery with standard diabetic doses of metformin 850 mg twice daily did not reduce tumor proliferation in women with endometrioid endometrial cancer awaiting hysterectomy [65]. With regard to combination therapy, twenty-one patients with advanced/refractory cancers received temsirolimus in combination with metformin. Of them, eleven patients had gynecological tumors, and 56% had stable disease as their best response. Overall, the combination therapy was well tolerated with modestly promising effectiveness [66].

### Thyroid cancer

Obesity has also been linked with an increased risk of thyroid cancer [67]. Metformin alone inhibits the invasion and metastasis of obesity-activated thyroid cancer in a mouse model, but not thyroid tumor growth. Metformin combined with JQ1, an inhibitor of the activity of the bromodomain-containing protein 4, suppressed thyroid tumor growth in the same mouse model [68]. In addition, metformin and the multikinase inhibitor sorafenib synergistically decreased the growth rate of anaplastic thyroid cancer cell lines and the outgrowth of derived cancer stem cells [69]. In a recent retrospective cohort study, these protective effects of metformin on thyroid cancer development, however, were observed especially in individuals with diabetes taking metformin for a longer duration or with a higher cumulative dose [70]. For obese patients or nondiabetic patients, the preventive and therapeutic role of metformin alone or in combination with other agents in thyroid cancer needs to be confirmed.

Different thyroid cancer cell lines have different susceptibilities to the antiproliferative effects of metformin [71, 72]. Metformin inhibits the secretion of CXCL8, which is associated with the growth and progression of tumors, in primary human normal thyroid cells and differentiated thyroid cancer cells [73]. Metformin was also reported to inhibit medullary thyroid cancer cell growth in a dose- and time-dependent manner and induce apoptosis [74]. The expression of mGPDH may predict susceptibility to the growth inhibitory effects of metformin *in vivo* [72]. Whether the effect of metformin on nondiabetic patients with different histological types of thyroid cancer is consistent with preclinical studies and the significant effect predictor need to be further investigated.

### Other cancers

In the era of immunotherapy and targeted therapy, the prognosis of patients with advanced melanoma has been significantly improved. Unfortunately, primary and secondary resistance to drugs is still observed, leading to treatment failure. Therefore, identifying new anti-melanoma agents is urgent. Metformin was reported to suppress the growth and motility of melanoma cells [75]. A pilot, prospective and multicenter study to investigate the effect of metformin (1000 mg three times daily) in patients with metastatic melanoma who progressed after first-line treatment and were not eligible or did not respond to ipilimumab was conducted, and it showed a lack of efficacy. The objective response rate in this study was 0%, as no patient obtained a CR or PR at 6 months [76]. It has also been reported that the metformin and paclitaxel combination as a second-line treatment was poorly tolerated in patients with gemcitabine-refractory advanced adenocarcinoma of the pancreas, with all patients presenting stable disease [77].

There is also increased interest in the use of metformin for glioblastoma. The phase 1 lead-in to a phase 2 factorial study showed that temozolomide plus memantine, mefloquine, and metformin are feasible and overall well tolerated as postradiation adjuvant therapy for newly diagnosed glioblastoma [78]. Currently, a phase 1b/2 clinical trial of metformin and chloroquine is recruiting patients with IDH1-mutated or IDH2-mutated solid tumors, including glioma [79].

Preclinical data showed that metformin inhibited proliferation and induced apoptosis in oral squamous cell carcinoma cells *in vitro* and *in vivo* [80]. A clinical trial with a small sample size conducted by Joseph et al. demonstrated that metformin has anticancer effects in head and neck squamous cell carcinoma by inducing apoptosis, altering stromal markers of metabolism and senescence and increasing immune infiltrate [81]. Furthermore, they found that apoptosis induced by metformin was greater in HPV-negative head and neck squamous cell carcinoma than in HPV-positive oropharyngeal squamous cell carcinoma [82]. Further research is necessary to assess the effect of metformin on the tumor microenvironment of head and neck squamous cell carcinoma.

In addition, metformin and 5-fluorouracil also showed overall modest activity in patients with refractory metastatic colorectal cancer in a phase 2 study. However, there was a trend for prolonged median survival for obese patients [83]. Results in two patients suggest that the combination of metformin and bromocriptine might be a new treatment for resistant prolactinomas, including one patient with impaired glucose tolerance [84].

## DISCUSSION

Despite the emergence of new anticancer drugs that can drastically alter the treatment paradigm and improve the outcomes of cancer, the considerable financial cost and the time required for new drug implementation are realistic problems that must be faced. Therefore, repurposing noncancer therapies with potential antitumor properties for cancer treatments offers a chance to improve survival while saving time and money. Given its low cost, favorable toxicity profile, and accumulating evidence regarding its anticancer effectiveness, metformin may have the potential to be a candidate in the last ten or more years.

**Figure 1:**
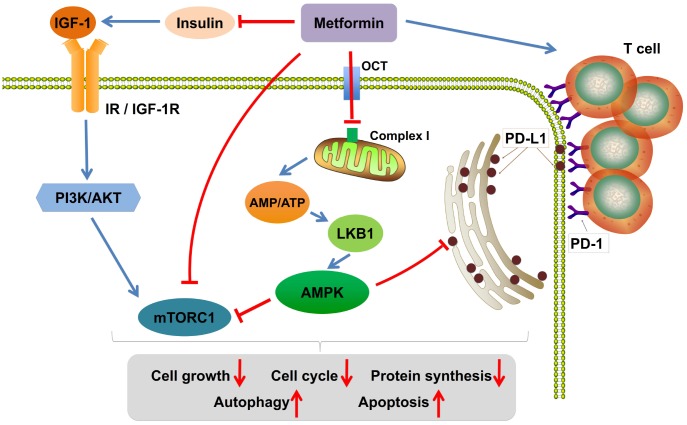
**Possible mechanisms of action of metformin in cancer therapy.** Metformin increases the ratio of AMP to ATP by inhibiting mitochondria complex I, activates the adenosine monophosphate activated protein kinase (AMPK) signaling pathway, and represses the insulin-like growth factor-1 receptor (IGF-1R) pathway. Furthermore, AMPK activation decreases the expression level of PD-L1, which allows cytotoxic T-lymphocyte-mediated tumor cell death. Last, metformin could increase the number of CD8+ T tumor-infiltrating lymphocytes. IGF-1, insulin-like growth factor-1; IGF-1R, insulin-like growth factor-1 receptor; IR, insulin receptor; LKB1, liver kinase B1; mTORC1, mammalian target of rapamycin complex 1; OCT, organic cation transporter; PI3K, phosphatidylinositol-4,5-bisphosphate 3-kinase; PD-1, programmed cell death protein-1; PD-L1, programmed death ligand-1.

The exact mechanisms of action of metformin are not clearly identified. It may influence different mechanisms depending on the way it uses. It can be used alone or combined with chemotherapeutic or targeted drugs [85, 86]. Regardless of the kind of usage, the most potent anticancer properties of metformin originate from activation of the LKB1-AMP-activated protein kinase (AMPK) signaling pathway. Metformin increases the ratio of AMP to ATP by targeting complex I of the mitochondrial respiratory chain, which leads to the activation of the upstream kinase LKB1 that phosphorylates and activates AMPK. AMPK activation can suppress mammalian target of rapamycin complex 1 (mTORC1), which plays a central role in cell growth, proliferation, and metabolism. In addition, metformin can also inhibit mitochondrial complex I or mTORC1 activity in an AMPK-independent manner [87]. Furthermore, metformin-induced activation of AMPK promotes PD-L1 phosphorylation, resulting in endoplasmic reticulum-associated PD-L1 protein degradation, which allows cytotoxic T-lymphocyte-mediated tumor cell death [88–90]. Possible mechanisms of action of metformin in cancer therapy are shown in Figure 1. Metformin has shown multiple target functions in terms of the mechanism of action. Its antitumor effect has also been confirmed by *in vitro* and *in vivo* experiments; however, the outcomes in the clinical trials, especially for nondiabetic patients with cancer, are not as satisfactory as expected. Not all individuals treated with metformin experience the clinical survival benefit, and some also develop poorly tolerated side effects. The possible reasons for these differences in efficacy and toxicity remain unclear.

One potential explanation is that the potential optimal dose, schedule, and duration are unclear. There are different usages of metformin in each clinical trial. For the prevention of cancer in nondiabetic patients mentioned above, the dosage of metformin varies from 250 mg/d, 850 mg/d, 500 mg twice daily to 1700 mg/d, and the diversities in the dose of metformin in the application of cancer treatment also exist. The duration of metformin also varies from clinical trial to clinical trial. For example, a relatively short dosing schedule was chosen in some trials to avoid affecting surgical management. A previous study showed that metformin inhibited proliferation and induced the apoptosis of tumor cells in a significant time- and dose-dependent manner. The dose-related tumor reduction was also confirmed in a mouse model, which potentially highlighted a dose-dependent component of the clinical effect of metformin overall [91]. In addition, it is also necessary to consider that the dose of metformin required to act on different pathways is different. For example, studies have shown that much higher concentrations of metformin are needed to exert its direct effects on the AMPK-mTOR pathway [91, 92]. In most cases, the doses of metformin used in preclinical studies *in vitro* and vivo are not comparable with doses achievable in clinical trials in humans, which may be 10–100 times higher than maximal serum levels of metformin achieved in humans [14, 93]. The effective concentration of metformin on target organs is probably one of the major obstacles for these unsatisfactory results [94]. It was reported that plasma levels of metformin were significantly higher after injection than oral administration in a mouse model [91]. Nonconventional routes of administration, such as inhalation for carcinomas of the lung or rectal suppositories for rectal cancer, may be an efficient channel to achieve short-term high-dose exposure in cancer tissues [94]. In addition, metformin at the dose commonly used in diabetes sometimes did not improve outcome in nondiabetic patients with cancer. However, few clinical trials were conducted that contained different metformin dose levels with dose escalation.

It is generally accepted that the adverse events increased by dose, and a high dose of metformin is associated with the risk of developing lactic acidosis and adverse gastrointestinal effects. Overall, the metformin-dosing schedule used was well tolerated, showing that long-term metformin treatment was associated with few adverse effects in nondiabetic patient populations. For some combination therapy, metformin was shown to be able to reduce doxorubicin-induced cardiotoxicity [95] and inhibit kidney uptake of peptidyl radiotracers, protecting the kidney from nephrotoxicity *in vivo* model [96], but there is a lack of evidence on clinical trials in nondiabetic patients. There are also a few cases of side effects that are poorly tolerated, such as everolimus combined with metformin in the treatment of advanced cancer [97]. It was reported that there was an increased rate of biochemical VitB12 deficiency after 6 months of metformin in nondiabetic breast cancer patients [98]. Although this was not associated with anemia, VitB12 monitoring in metformin-treated individuals should be implemented [99].

The heterogeneity of histological subtypes and genotypes among the patients with cancer might have also contributed to the different clinical benefits. This point is supported by the observation that metformin induced significant apoptosis only in the small cell carcinoma cell line but not in other human lung cancer cell lines including squamous, adeno-, and large cell carcinomas. Metformin and cisplatin might also be partly antagonistic in various histological subtypes of human lung cancer cell lines but not in the adenocarcinoma cell line [100]. The sensitivity of two cell lines of clear cell renal cell carcinoma to metformin was also shown to be different [101].

Moreover, it has been demonstrated that not only the histological stratification can differ but also the gene mutations encountered in tumors can affect the response as well. Cancer cells exhibit various mutations. The expression of K-ras is notably increased in as many as one-third of all tumors. Researchers noticed that metformin induced apoptosis and inhibited cell proliferation in K-ras mutant tumors but not in K-ras wild-type tumors [102]. Apart from K-ras gene mutation, other genetic alterations, including p53, LKB1 and phosphatidylinositol 3-kinase (PI3K), may also impact the anticancer efficiency of metformin [103]. Different genotypes may also have different impacts on the response to combination therapy. It was reported that metformin in combination with pemetrexed significantly altered the cell cycle distribution of a certain adenocarcinoma cell line [104]. Tumor genetic profiling is required to identify patients most likely to benefit from metformin treatment. In addition, the efficiency of metformin may also be influenced by BMI and whether there is insulin resistance in nondiabetic patients. Most studies indicated that metformin is most effective in patients with high BMI and insulin resistance. Nonetheless, further testing is needed to determine the optimal levels of metformin required to maximize benefits in nondiabetic patients and at that dose, which molecular effects and gene expression changes are predominant.

Recently, metformin was shown to be able to modulate the interaction between tumor cells and their microenvironment and to have an immune-mediated antitumor effect. Metformin can enhance antitumor immunity by many approaches, thereby affecting antitumor T cell generation, antitumor T cell effector function and the formation of T cell memory [88, 105–108]. Immunotherapy has become one of the most important breakthroughs in cancer treatment. Immune checkpoint inhibitors have been demonstrated to enhance antitumor immune responses by the recovery of T cell function [109, 110]. A retrospective chart review study conducted by Keisuke Shirai et al. showed that there is an overall trend towards better outcomes in patients receiving ipilimumab, nivolumab, and/or pembrolizumab plus metformin [111]. A recent study by Han et al. demonstrated that metformin reversed PARP inhibitor-induced epithelial-mesenchymal transition (EMT) and PD-L1 expression, which sensitized PARP inhibitor-resistant cells to cytotoxic T cells, suggesting that the combination may increase tumor sensitivity to immunotherapy [112]. Currently, numerous clinical trials involving metformin and immune checkpoint inhibitors in nondiabetic cancer patients are active around the world. Information was obtained from a service of the United States National Institutes of Health (http://clinicaltrials.gov/). Ongoing clinical studies of metformin and immune checkpoint inhibitor combination therapy in nondiabetic patients with cancer are summarized in Table 1. Thus, promising outcomes might be achieved soon.

**Table 1:** Summary of ongoing clinical studies of metformin and immune checkpoint inhibitors combination therapy in nondiabetic patients with cancer.

**No.**	**NCT Number**	**Title**	**Study design**	**Enrollment**	**Status**	**Diseases**	**Interventions**	**Sponsor**	**Start date**
1	NCT03994744	Assessing Safety and Efficacy of Sintilimab and Metformin Combination Therapy in SCLC	Phase 2 Open Label	68	Recruiting	ED-stage SCLC patients resistant to or relapsed after standard chemotherapy	PD-1 inhibitor Sintilimab plus Metformin	Hunan Cancer Hospital, China	20-Aug-2019
2	NCT03800602	Nivolumab and Metformin in Patients With Treatment Refractory MSS Colorectal Cancer	Phase 2 Open Label	28	Recruiting	MSS stage IV colorectal cancer	Nivolumab plus Metformin	Emory University Hospital, Emory Saint Joseph's Hospital, United States	15-Jan-2019
3	NCT03618654	Durvalumab With or Without Metformin in Treating Participants With Head and Neck Squamous Cell Carcinoma	Phase 1 Randomized Open Label	38	Recruiting	Head and neck squamous cell carcinoma	Durvalumab vs Durvalumab plus Metformin	Sidney Kimmel Cancer Center at Thomas Jefferson University, United States	1-Nov-2018
4	NCT03311308	A Trial of Pembrolizumab and Metformin Versus Pembrolizumab Alone in Advanced Melanoma	Phase 1 Non-Randomized Open Label	30	Recruiting	Advanced Melanoma	Pembrolizumab vs Pembrolizumab plus Metformin	University of Pittsburgh Medical Center Hillman Cancer Center, United States	6-Dec-2017
5	NCT03048500	Nivolumab and Metformin Hydrochloride in Treating Patients With Stage III-IV Non-small Cell Lung Cancer That Cannot Be Removed by Surgery	Phase 2 Open Label	51	Recruiting	Recurrent or Stage III-IV NSCLC	Nivolumab plus Metformin	Northwestern University, United States	6-Jun-2017
6	NCT04114136	Anti-PD-1 mAb Plus Metabolic Modulator in Solid Tumor Malignancies	Phase 2 Open Label	108	Not yet recruiting	Solid Tumor Malignancies	Anti-PD-1 mAb (nivolumab or pembrolizumab) plus Metformin vs Anti-PD-1 mAb (nivolumab or pembrolizumab) plus Rosiglitazone	UPMC Hillman Cancer Center, United States	15-Oct-2019

Abbreviation: SCLC: small cell lung cancer, ED-stage: extensive stage disease, PD-1: programmed cell death protein 1, MSS: microsatellite stable, NSCLC: non-small-cell lung cancer.

## CONCLUSIONS

Metformin is an inexpensive drug with an excellent safety profile, but its potential anticancer effects in nondiabetic patients with cancer are controversial. Several studies report a trend toward decreasing the incidence of several cancers. Improved outcomes were also demonstrated in nondiabetic cancer patients with a certain histological subtype or genotype who were treated with metformin alone or in combination with another therapy. However, the reported results of prospective and randomized trials are limited. A large number of clinical trials are ongoing, and we are looking forward to promising outcomes to improve the management of nondiabetic cancer patients. Moreover, as the immune property of metformin was investigated, further studies of the immunomodulatory effect of metformin on cancer cells should also be taken into account to optimize its clinical use.
